# Supplementary material for: New hypotheses of cell type diversity and novelty from orthology-driven comparative single cell and nuclei transcriptomics in echinoderms
Source: eLife. 2023 Jul 20;12:e80090. doi: 10.7554/eLife.80090 (PMC10396339; doi:10.7554/eLife.80090)
Supplement: Supplementary file 5. [file elife-80090-supp5.pdf]

## A single nucleus atlas of early development in *S. purpuratus*

As a proof of concept, we performed single-nucleus RNA-seq on *S. purpuratus* embryos to see if we could create biologically meaningful clusters that correspond to known cell types. We sample 4 time points spanning the blastula through the mid-gastrula stage (6hpf, 15hpf, 23hpf, and 33hpf)

Of particular note is that our final clustering resulted in 3 PMC clusters, whereas previous whole-cell work has only yielded 1 PMC cluster without conducting any sub clustering analysis (Paganos et al. 2021; Foster et al. 2020). Cluster 13 expresses aristaless-like homeobox (*alx1*), AT-rich interactive domain-containing protein 3A (*arid3a*, previously called dead ringer homolog), and SRY-box transcription factor 4 *(sox4)*, genes in the most upstream, an early-acting portion of the PMC GRN. Cluster 11 is marked by the expression of vascular endothelial growth factor receptor 1 *(flt1,* also known as *VEGFR1*), a gene previously implicated in activating biomineralizing effector genes, as well as mesenchyme-specific cell surface glycoprotein (*msp130*), a gene present in the biomineralization module of the PMC GRN. Cluster 14 is characterized by the expression of other genes involved in the process of biomineralization, including spicule matrix protein SM37 (*sm37*), and spicule matrix protein SM37 (*sm37*). The distinction of our PMC clusters, consistent with the order of GRN deployment suggests that nuclei give us an advantage for detecting subpopulations of this morphologically complex cell type.

**Pigment cells** were marked by the expression of Zic family member 1 (*zic1*), glial cells missing transcription factor-like protein (*gcml*), and GATA binding protein 6 (*gata6*). It also shows broad expression of probable polyketide synthase 1 (*pks1*).

The **Oral NSM** is characterized by the expression of known blastocoel cell markers like RUNX family transcription factor 2 (*runx2*), and prospero homeobox 1 (*prox1*). This identity is further supported by the expression of genes related to immune function, including complement C3 (*c3*), scavenger receptor cysteine-rich protein variant 2 (*srcr2*), TEK receptor tyrosine kinase (*tek*) and DD186 protein, upregulated upon bacterial challenge (*dd186*).

**Cluster 5** was annotated as endoderm because it  is clearly distinguished by the expression of PR/SET domain 1 (*prdm1*), homeobox protein TGFB induced factor homeobox 2-like (*tgif2l*), and calcium-binding protein (*endo16*)

**Cluster 4** corresponds to the ectodermal and endodermal veg1 region of the embryo is marked by the expression of protein DVR-1 homolog (*dvr1*) and transcription factor ETS transcription factor ELK 1 (*elk1*).

**Cluster 3 Ciliary band** is characterized by the expression of homeobox protein SIX homeobox 6 (*Sp-six6*), polycystin 2, transient receptor potential cation channel (*pkd2*), and EPH receptor A2 (*epha2*).

The **oral ectoderm** is marked by the strong expression of chordin (*chrd*) as well as H6 family homeobox-like 2 (*hmxl2*).

**Clusters 8 and 0** are annotated as aboral ectoderm and are characterized by the expression of T-box transcription factor 2 (*tbx2*), SAM pointed domain-containing ETS transcription factor (*spde*f, formerly known as *Ets4*), iroquois homeobox 4 (*irx4*), Kruppel like factor 6 (*klf6*), spec 1a protein (*spec1a*), and spec 1a protein (*spec2c_1*).

Three neural clusters were identified in our single nucleus atlas. **Cluster 6** was annotated as endocrine-responsive neural because this  cluster expresses a variety of transcription factors involved in neural differentiation, including SRY-box transcription factor 2 (*sox2*), SRY-box transcription factor 14 (*sox14*), H2.0-like homeobox protein (*hlx*), and glial cells missing transcription factor-like (*gcml*). This cluster also expresses genes linked to hormone signaling, estrogen related receptor (*err*), allatostatin-A receptor (*AstAR*), and membrane-associated progesterone receptor component 1-like  (*PGRMC1L*).

**Cluster 2** was identified as dopaminergic neurons based on the marker gene activity of D(1) dopamine receptor (*DRD1*) and ankyrin containing gene specific for apical tuft 1 (*ankat1*).

**Cluster 9** is also annotated as neural-based on the expression of neuroblast differentiation-associated protein AHNAK (*AHNAK)* and *neurabin-1* and is specific neuronal lineage cannot be determined based on the marker genes expressed at these stages of development.

Only cluster 1 was not able to be annotated, as it expressed no previously-characterized cell type markers.

The ability to resolve these diverse cell stages at such an early point in development, despite the small size of the dataset, demonstrates the strengths of a single nucleus approach to developmental atlas creation.
