# Supplementary material for: New hypotheses of cell type diversity and novelty from orthology-driven comparative single cell and nuclei transcriptomics in echinoderms
Source: eLife. 2023 Jul 20;12:e80090. doi: 10.7554/eLife.80090 (PMC10396339; doi:10.7554/eLife.80090)
Supplement: Supplementary file 6. [file elife-80090-supp6.pdf]

Detailed cluster annotation for *P. miniata* sn-RNA-seq

Cluster 0 had only two genes in its marker set (*nuclear receptor subfamily 0 group B member 1-like* and *filamin-B*) and we therefore overall had low confidence in our ability to annotate this cluster. We characterized this cell cluster as **mesoderm-like** based on absence of markers. That is, key endoderm (*gata6, arid3a*, T-box transcription factor 2 (*tbx2*)) and ectoderm (ets variant transcription factor 6 (*etv*), *PM-onecut2*) underexpressed in cluster 0 compared to other clusters.

Cluster 1 we annotated as **ectoderm** based on the presence of the one cut domain family member 2-like (*Pm-onecut2*) , goosecoid homeobox-like (*gscl*), and forkhead box j1 (*foxj1*), all of which are known to be expressed in *P. miniata* ectoderm (Yankura et al. 2010) .

Cluster 2 was annotated as **ectoderm**, based on presence of the marker genes, *Pm-onecut2* and orthodenticle homeobox 2 (*otx2*). *Foxj1* and *gscl* were also expressed. This cluster was therefore similar to cluster 1, with significant expression of *otx2*, and higher levels of expression of *Pm-onecut2* making it distinct from cluster 1.

Prdm1 (formerly known as *Blimp1/krox*) which is expressed in the veg1 ectoderm/endoderm boundary is a marker gene of **cluster 3**. We also detected low levels of *gata6, tgif2l, foxa1,* and *foxn3* which are all expressed in the endoderm. We therefore annotated cluster 3 as **veg1 ectoderm/endoderm cells**.

Cluster 4 was annotated as **post-oral ciliary band ectoderm**. A marker of this cluster is *foxj1* which is broadly expressed in the ectoderm. *Nk1-2l* and *gscl* also show low levels of expression in this cluster and are specifically expressed in the post-oral ciliary band. Additionally, multiple cytoskeletal and motor protein associated genes appear in this clusters marker gene set, including stabilizer of axonemal microtubules 1-like (*saxo1*), stabilizer of axonemal microtubules 2 (*saxo2*), and dynein heavy chain 2, axonemal-like (*DNAH2L*), and *tectin b1*.

The **ventral ectoderm** expressed gene *tbx2* which is a strong marker gene in **cluster 5** We also noted expression of mothers against decapentaplegic homolog 6-like (*smad5l*) as a known marker of ventral ectoderm in sea urchins. It is also marked by genes relating to ciliary band function like cilia and flagella associated protein 410 (*CFAP410*)and dynein heavy chain 2, axonemal-like (*DNAH2*). This cluster was here annotated as ventral ectoderm

Cluster 6 was annotated as muscle/coelom. It was marked by the expression of myosin light chain kinase smooth muscle-like (*MLCKL*). Secreted frizzled-related protein 5-like (*SFRP5L*), Zic family member 1 (*zic1)*, homeobox protein *SIX6-like (Six6L)*, and *ets1* are also lowly expressed in this cluster. *Frzl-5* is expressed in the anterior coelom, the source of muscle cells.

Cluster 7 was marked by the expression of chordin-like (*chrdl*), *gscl, arid3a*, and transcription factor AP-2 alpha *(tfap2a*). In situ hybridization (Fig 7.D.vi) has shown *tfap2a* localizes to the oral ectoderm. We annotated this cluster as **oral ectoderm**.

Cluster 8 shows expression of multiple veg1 **ectoderm** genes, including Wnt family member 3 (*wnt3*) and transcription factor HES-4 (*HES4*). The presence of NK1 transcription factor-related protein 2-like (*nk1-2l*) is also detected. The gene lim homeobox 1 (*lhx1*) is highly expressed in its marker gene set and *lhx1* is also expressed in the ectoderm of *S. purpuratus*. Based on this, we determined cluster 8 is **veg1 ectoderm cells.**

Like cluster 7, **cluster 9** has *tfap2a* and *arid3a* in its marker gene set. In comparison to cluster 7, cluster 9 includes *epha2* as a marker gene, which is expressed in ciliary band (Krupke and Burke 2014). Therefore we also annotate **cluster 9 as oral ectoderm.**

Cluster 11 was annotated as **veg1 ectoderm/endoderm**. *Hbox 7, gata6, Wnt3*, and *prdm1* are in this cluster's marker gene set, all of which are in the *P. miniata* veg1 endoderm/ectoderm GRN. Cluster 11 was distinct from clusters 3 and 8 based on the maker gene *wnt1* and strong expression of *hbox7.*

Cluster 12 was not marked by the expression of any genes with pre-existing literature in the sea star and thus we were unable to assign it an identity. It remains **undetermined**. Its top 3 marker genes that have been named are organic cation transporter protein-like (*Orctl)*, probable beta-D-xylosidase 6 (*BXL6*), and poly [ADP-ribose] polymerase tankyrase-like (*TNKS2L*).

Cluster 13 has *gata6, prdm1, foxn3,* and *tgif2l* in its marker gene set, all of which are part of the *P. miniata* endoderm GRN. Compared to cluster 3, this higher expression of *gata6* and presence of *wnt3. Wnt3* is strongly expressed in endoderm (McCauley et al. 2013). Thus, this cluster is defined as **endoderm.**

Cluster 15 has hedgehog family (*hh*) and fibroblast growth factor receptor (*fgfrl1*) in its marker gene set, which are both expressed in the foregut . Smoothelin-like (*SMTN*L) and forkead box A1 (*foxa*) are also expressed in this cluster. Therefore, we define cluster 15 as the **foregut**.

Cluster 19 is marked by the expression of s*ox2, sox14*, and several cell cycle regulators, including geminin-like (*GMNNL*), PCNA-associated factor-like (*PCLAFL*), chromatin licensing and DNA replication factor 1 (*cdt1*), cyclin A2 (*ccna2*), and cyclin B2 (*ccnb2*). Therefore, we define cluster 19 as **dividing ectoderm.**

Cluster 20 is marked by the expression of *foxj1*, several genes relating to cilia and flagella activity. These include members of the tektin family (tektin 2 (*tekt2*), tektin 3 (*tekt3*), and tektin 4 (*tekt4*)), and radial spoke head component 1 (*rsph1*). We, therefore, annotate this cluster as broad **ciliary band.**
